# Comparison of Relative Change with Effect Size Metrics in Alzheimer’s Disease Anti‐amyloid Clinical Trials

**DOI:** 10.1002/alz.094862

**Published:** 2025-01-09

**Authors:** Terry E. Goldberg, Seonjoo Lee, Davangere Devanand, Lon S. S. Schneider

**Affiliations:** ^1^ Columbia University Medical Center, New York, NY USA; ^2^ New York State Psychiatric Institute, New York, NY USA; ^3^ Taub Institute for Research in Alzheimer’s Disease and the Aging Brain, Columbia University, New York, NY USA; ^4^ Mailman School of Public Health, Columbia University, New York, NY USA; ^5^ Columbia University Irving Medical Center, New York, NY USA; ^6^ Department of Psychiatry and Behavioral Sciences, Keck School of Medicine, University of Southern California, Los Angeles, CA USA

## Abstract

**Background:**

Percent slowing of decline is frequently used as a metric of outcome in Alzheimer’s disease (AD) clinical trials, but it may be misleading. Our objective was to determine whether percent slowing of decline or Cohen’s d is the more valid and informative measure of efficacy.

**Method:**

Outcome measures of interest were percent slowing of decline; Cohen’s d effect size, and number‐needed‐to‐treat (NNT). Data from a graphic were used to model the inter‐relationships among Cohen’s d, placebo decline in raw score units, and percent slowing of decline with active treatment. NNTs were computed based on different magnitudes of d. Last, we tabulated recent AD anti‐amyloid clinical trials that reported percent slowing and for which we computed their respective d’s and NNTs.

**Result:**

We demonstrated that d and percent slowing were independent. While percent slowing of decline was dependent on placebo decline and did not include variance in its computation, d was dependent on both group mean difference and pooled standard deviation. We next showed that d was a critical determinant of NNT, such that NNT was uniformly smaller when d was larger. In recent AD associated trials including those focused on anti‐amyloid biologics (aducanumab, lecanemab, gantenerumab, and donanemab), d’s were below 0.23 and thus small, while percent slowing was in the 12%‐27% range and NNTs ranged from 14‐34.

**Conclusion:**

Standardized effect size is a more meaningful outcome than percent slowing of decline because it determines group overlap, which can directly influence NNT computations, and yield information on the likelihood of minimum clinically important differences. In AD, greater use of effect sizes, NNTs, rather than relative percent slowing, will improve the ability to interpret clinical trial results and evaluate the clinical meaningfulness of statistically significant results.

